# Feasibility of a Stop Smoking Program for Healthcare Workers in an Italian Hospital: Econometric Analysis in a Total Worker Health® Approach

**DOI:** 10.5334/aogh.4153

**Published:** 2023-08-30

**Authors:** Reparata Rosa Di Prinzio, Giorgia Bondanini, Federica De Falco, Maria Rosaria Vinci, Vincenzo Camisa, Annapaola Santoro, Gabriele Arnesano, Guendalina Dalmasso, Massimiliano Raponi, Eugenio Di Brino, Americo Cicchetti, Nicola Magnavita, Salvatore Zaffina

**Affiliations:** 1 Alta Scuola di Economia e Management dei Sistemi Sanitari (ALTEMS), Università Cattolica del Sacro Cuore, Rome, Italy; 2 Occupational Medicine Unit, Bambino Gesù Children’s Hospital IRCCS, Rome, Italy; 3 Department of Human Science, European University of Rome, Italy; 4 Post-Graduate School of Occupational Health, Università Cattolica del Sacro Cuore, Rome, Italy; 5 Health Directorate, Bambino Gesù Children’s Hospital IRCCS, Rome, Italy; 6 Occupational Health Unit, Department of Woman, Child & Public Health, A. Gemelli Policlinic Foundation IRCCS, Rome, Italy

**Keywords:** workplace health promotion, smoking cessation, sickness absence, econometric analysis, workplace, occupational health, break-even analysis

## Abstract

**Background:**

Over 20% of healthcare workers (HCWs) are active smokers. Smoking is a targeted issue for workplace health promotion (WHP) programs.

**Objective:**

Our study aims to evaluate the effectiveness of the Stop Smoking Promotion (SSP) intervention, a 6-hour training course for HCWs, which took place from May 2018 to July 2019.

**Methods:**

We compared HCWs who successfully quit smoking (n = 15) to those who did not (n = 25) in terms of Sickness Absence Days (SADs). Moreover, we conducted an econometric analysis by calculating the return on investment and implementing a break-even analysis.

**Findings:**

Among the 40 enrolled workers, a success rate of 37.5% was observed after a span of over two years from the SSP intervention (with nurses and physicians showed the best success rate). Overall, participants showed a noticeable absenteeism reduction after the SSP intervention, with a reduction rate of 85.0% in a one-year period. The estimated ROI for the hospital was 1.90, and the break-even point was 7.85. In other words, the organization nearly doubled its profit from the investment, and the success of at least eight participants balanced costs and profits.

**Conclusion:**

Our pilot study confirms that WHP programs are simple and cost-saving tools which may help improve control over the smoking pandemic in healthcare settings.

## Introduction

Tobacco smoking represents the world’s leading cause of preventable non-communicable diseases, causing around 8 million deaths each year [1]. Globally, 19% of adults are current smokers (men 33%; women 6%) [1]. The burden of tobacco use extends to individuals exposed to second-hand smoke and those using smokeless tobacco products, comprising at least five million users for each WHO region, particularly prominent in low-income countries [2]. Tobacco use carries many severe adverse health consequences, including lung and heart diseases, chronic respiratory conditions, cancer, and diabetes [3], and endangers children’s regular growth [4]. Among working adults, one in five workers is a smoker [5]. Among industries, construction workers exhibit the highest smoking prevalence, surpassing 34%, while those in education services have the lowest prevalence at 11% [5]. According to recent data, more than 20% of healthcare workers (HCWs) are active smokers [6]; in Italy, this percentage increases to 23% on average [7]. Moreover, smoking has been associated with an increased absenteeism rate [8], and with an increase of medical errors and occupational injuries [9].

Many strategies have been developed for quitting smoking. Individual and group counselling, pharmacological treatment to overcome nicotine addiction, and combined interventions have been found to be more effective than self-help interventions and social support alone [10]. Comprehensive smoke-free policies aimed at providing environments without exposure to tobacco smoke, assure an improvement of health, productivity, and social cohesion [4]. Furthermore, these policies protect non-smokers from the dangers of passive smoking and encourage smokers to quit or reduce consumption [11].

Work environments influence smoking behaviors; high job demands were associated with increased smoking, whereas social support was correlated to quitting [12]. Smoking habits have long been a targeted issue for workplace health promotion (WHP) programs [13]. Various approaches have been developed to promote smoke cessation in the workplace, such as proactive telephone counselling [13,14]. Several studies have shown how courses offered in the workplace regarding the promotion and cessation of smoking have positively influenced employees [15,16]. Carrying out these courses in the workplace offers several benefits, including access to a large workforce, reaching a very diverse audience, and encouraging healthy behaviors for the active segment of the population who find it challenging to visit hospitals [16]. It has been found that smoking cessation interventions delivered during work hours provide high levels of abstinence [17]. Although the incentives offered by employers have shown an improvement in smoking cessation rates at long-term follow-ups [18], the total number of workers who quit is low [10]. This issue particularly affects the healthcare sector, whose workers are invested in a significant ethical responsibility for users and for society [19]. Healthcare organizations, which assume that their employees lead healthy lives, underestimate the impact of specific interventions on the issue. As a result, organizations’ efforts to combat the high rate of active smoking among HCWs have waned with time, and ineffective solutions (such as training programs) have been phased out [19]. By pointing out the notable increase in productivity brought on by targeted programs for quitting smoking, this paradigm could be changed.

Therefore, in this paper we aim to fill this gap through a description of the *“Stop Smoking Promotion”* (SSP) intervention designed for the hospital’s active smoker workforce. Our study aims to evaluate the effectiveness of the SSP intervention in terms of success rate. An econometric analysis is conducted by calculating the return on investment and implementing a break-even analysis for the program.

## Materials and Methods

### The “Stop Smoking Promotion” (SSP) intervention

The Health Surveillance Service of the Bambino Gesù Children’s Hospital in Rome has adopted for many years the principle of associating health promotion with occupational risk prevention, according to a model of workplace health promotion embedded in medical surveillance [20] which corresponds to the integration model known in the USA as Total Worker Health ® [21]. In this perspective, the SSP intervention has been set up as a part of the WHP plan. The physicians and psychologists of the service conducted an analysis of different intervention models reported in the literature, and decided to conduct a pilot study with a method that offered a priori good prospects for effectiveness.

The course program was based on the *“Easyway”* technique theorized by Allen Carr, and no drug administration was provided [15,16,17]. According to cognitive psychology strategies, participants were invited to restructure their personal beliefs about smoking. They were encouraged to consider the emotional abuse of nicotine dependence resulting from the smoking habit. The course did not focus on the well-known negative health effects of smoking; instead, it focused on the psychological dependence it creates. Participants were asked to visualize themselves in different scenes of common daily living, as proposed by the coach. The few interactions in the group were guided by the coach, and each participant focused on his/her perceptions. The course’s core mechanism revolves around the expectancy challenge, which is employed to lower the perceived benefits of smoking and thereby facilitates smoking cessation. Increasing self-efficacy expectations gives better control over this process [22].

For the sake of timeliness, we decided to conduct the first test with workers who were already planning to quit smoking. A call for quitting smoking was published in the landing page of the hospital addressed to all employees (n = 2800, with a prevalence of active smokers of 21%). The first 49 subjects who adhered to the courses by mailing to the hospital’s Occupational Health Service were enrolled for the pilot editions of the SSP program. Five editions of a six-hour training course were organized from May 2018 to July 2019. All 49 subjects participated in the course.

### Design of the study

A pilot observational longitudinal pre-post intervention study was set for HCWs who attended the SSP courses. After the course, a follow-up period was planned to investigate the success rate after six months, one year and two years. Considering that nine participants dropped out due to leaving the company during follow-up, 40 HCWs were included in the study (81.6%). Participants were mostly female (n = 28, 70.0%) and the mean age was of 44.63 years (SD: 10.41). Occupational data including seniority, shift work, and professional category were collected from the medical records of the periodical occupational surveillance; current information at the time of the SSP course was present in the last medical visit attended by participants. Econometrical data was made available by the hospital’s Human Resources Directorate.

Participants were divided into two groups, considering those who succeeded in quitting smoking (“non-smokers”), and those who did not (“smokers”), categorized at the end of the two-year follow-up. Non-smokers and smokers were compared in terms of seniority, professional category, shift work, sickness absence days (SADs), and annual work performance. SADs were calculated before and after the SSP program in the periods of 6 months, 12 months, and 24 months. Work performance data related to the annual evaluation were collected by the HCW’s direct superior. In anticipation of the possible loss of observations due to workers’ mobility to other health care providers or termination of employment, we planned to check the status of those exiting the cohort through a telephone interview. Due to heterogeneity with other workers, however, we did not include these cases in the cohort, and considered them separately.

### Statistical analyses

A descriptive analysis of demographic characteristics was carried out using mean and standard deviation for continuous variables and frequencies for categorical variables. Success rates of the SSP intervention were expressed in percentages. After ascertaining that data were not normally distributed using Kolmogorov-Smirnov normality test, non-parametric tests were used to compare the two groups. Smokers and non-smokers were compared using the Mann-Whitney U test and the Kruskal-Wallis test for demographic data, whereas the Wilcoxon signed rank test was applied to compare SADs paired pre-post intervention data in the whole sample and in smokers and non-smokers separately. Two-tailed *p* value < .05 was considered statistically significant. Data were analyzed using IBM Statistics Package for Social Sciences (SPSS) (version 26.0).

### Econometric analyses

The return on investment is defined as the ratio between net profit (difference between the gross profit and the invested capital) and invested capital [23]. For this purpose, the invested capital was computed as the sum of per capita costs for the training courses, whereas the gross profit was the recovered time from smoking during the working day. In this regard, only non-smokers were included in the economic exploitation of the recovered time from smoking: the mean number of cigarettes during the working hours (obtained from the referred number of cigarette/day) was multiplied by six minutes (the average time to smoke a cigarette) [23]. Then, the economic enhancement of recovered mean hours of non-smokers was used to perform the break-even analysis in order to determine the volume of activity from which the SSP intervention would have become profitable [24].

### Ethical considerations

The study was conducted according to the guidelines of the Declaration of Helsinki, and was approved by the Institutional Review Board of Bambino Gesù Children’s Hospital IRCCS (protocol code: Di Prinzio Reparata Rosa-RAP-2023-0003; date of approval: 09 February 2023). As established by the Italian legislation regarding obligatory occupational surveillance and privacy management, confidentiality was safeguarded. Informed consent was obtained from all participants enrolled in the study.

## Results

### Demographic characteristics

On average, seniority was 15.74 years (SD: 13.65). Participants were mainly nurses (n = 16; 40.0%), followed by administrative personnel (n = 14; 35.0%), technicians and biologists (n = 9; 22.5%), and physicians (n = 1; 2.5%). The majority of the participants worked shifts (n = 25; 62.5%). The professional category distribution of the sample traced the prevalence of active smokers in the overall hospital’s population. In fact, the prevalence of smokers among technicians and doctors was less than the prevalence we found in nurses and administrative personnel. Therefore, the sample can be considered as representative of our population.

### Success rate

After six months, a total of 21 participants (52.5%) quit smoking. This rate decreased to 40.0% after 12 months and finally to 37.5% after 24 months (n = 15). Smokers and non-smokers did not noticeably differ in terms of sex and age, seniority, or shift work. Conversely, a different success rate was identified among professional categories. The nurses and physician showed the major success rate (64.7%), followed by administrative personnel (21.4%) (Table 1).

**Table 1:** Comparison between smokers and non-smokers after two years from the SSP intervention using the Mann-Whitney U test and Kruskal-Wallis test.


	SMOKERS VS. NON-SMOKERS	P VALUE

Age	45.68 ± 10.35 vs. 42.73 ± 10.45	0.391

Seniority	17.17 ± 13.56 vs. 13.47 ± 13.94	0.558

Sex		

Male	7 (58.3%) vs. 5 (41.7%)	0.783

Female	18 (64.3%) vs. 10 (35.7%)	

Shift work	14 (56.0%) vs. 11 (44.0%)	0.376

Professional category *	25 (62.5%) vs. 15 (37.5%)	0.008

Administrative personnel (1)	11 (78.6%) vs. 3 (21.4%)	

Technicians and biologists (2)	8 (88.9%) vs. 1 (11.1%)	

Nurses and physician (3)	6 (35.3%) vs. 11 (64.7%)	

Comparison (3) vs (1)		0.043

Comparison (3) vs (2)		0.024


*Notes*: * Kruskal-Wallis test.

Nine dropouts were mainly females (n = 8, 88.9%), administrative workers (n = 6, 66.6%) and nurses (n = 3, 33.3%). They did not differ in terms of age, sex or seniority compared to the overall sample. They dropped out from the study at the first follow-up at six months. However, they were contacted for the final interview on the success rate; most of them answered that they succeeded to stop smoking (n = 8, 88.9%).

### SADs before and after SSP Intervention

Overall, participants showed a noticeable absenteeism reduction after SSP intervention, with a reduction rate of 85.0% in absenteeism in the one-year period (Table 2). The difference became more statistically relevant after two years. Work performance did not significantly differ between years in the overall sample.

**Table 2:** Pre vs. post SADs comparison after 6 months, 12 months, and 24 months from the SSP course in the whole sample and in smokers and non-smokers using the Wilcoxon signed rank test.


	SADS PRE-SSP VS. POST-SSP(MEAN ± SD)	P VALUE

**Overall**		

**6 months**	0.5 ± 1.5 vs. 0.1 ± 0.0	0.043

**12 months**	1.3 ± 2.7 vs. 0.2 ± 1.1	0.036

**24 months**	2.7 ± 4.6 vs. 0.49 ± 1.5	0.010

**Smokers**		

**6 months**	0.5 ± 1.4 vs. 0.2 ± 0.0	0.109

**12 months**	1.5 ± 2.7 vs. 0.3 ± 1.4	0.092

**24 months**	3.1 ± 4.9 vs. 0.8 ± 2.0	0.038

**Non-smokers**		

**6 months**	0.7 ± 2.1 vs. 0.1 ± 0.0	0.180

**12 months**	0.9 ± 2.9 vs. 0.2 ± 1.1	0.180

**24 months**	1.5 ± 3.7 vs. 0.8 ± 2.0	0.131


*Notes*: SADs: sickness absence days; SSP: Stop Smoking Promotion.

### Econometric Assessment

Each participant used to smoke five cigarettes during a work shift (median value), thus saving 30 minutes (median value, IQR: 21.88–35.00) on average. The cost of the training course was € 330.00 per capita, which accounted for € 13,200.00 (global invested capital). Considering the average hourly cost for a HCW (€ 24.26), the gross profit was € 38,134.25 in a year. Thus, the estimated ROI for the hospital was 1.90. The break-even point was 7.85 (Table 3).

**Table 3:** Economic evaluation of the SSP intervention.


	ECONOMIC EVALUATION	CRITERIA

**Invested capital**	€ 13,200.00	Per capita cost of the training course: € 330,00

**Gross profit**	€ 38,134.25	Weighted sum of the per capita recovered time in a year, considering the mean cost of a working day: € 169,80 and the mean time of six minutes per cigarette

**Net profit**	€ 24,934.25	Difference between the gross profit and the invested capital

**ROI**	1.90	Ratio between the net profit and the invested capital (considering the one-year period)

**Pre-SSP situation**	€ 1,682.45	Mean hours lost for smoking during work shifts

**Post-SSP situation**	€ 0.00	Recovered mean hours (from non-smokers)

**BEP**	7.85	The number of participants who quit smoking at which the costs of the SSP program would equal the profit


## Discussion

This study showed a success rate of 37.5% for the SSP intervention after two years. Success was significantly related to the professional category with nurses and physicians being the most successful category. ROI was computed as 1.90 and BEP was 7.85. In other words, the organization had almost double the profit for the investment, and the success for at least eight participants balanced costs and profits.

Several methods have been found as effective to stop smoking, including written advice, individual counseling, telephonic counseling, group courses, medical treatment based on nicotine-replacement therapy, bupropion, and nortriptyline [25]. Standing that the success rate of smoking cessation is real when a subject manages a nicotine-free status for at least six months from interruption [18], our results highlighted a greater success rate than that reported in most previous studies on the same method. In fact, an effectiveness of 23% after six months [22], 22% after twelve months [22], and 19.4% after twenty-six weeks [17,18] has been shown for the method implemented. In Austria, one-year results were found comparable to our findings, achieving a success rate of 40–55% [26]. Additionally, a long-term study on a three-year time frame showed the highest success rate documented in literature (51.4%) [27]. The course we adopted had two essential characteristics of successful WHP interventions: a short duration and a group-based technique. Alongside to the individual motivational factor [28], these qualities make them a feasible opportunity during working hours and contribute to create peers’ social support, therefore rising the course’s long-term efficacy [16,29]. The social working environment has been identified as a significant determinant of success in quitting smoking [30]. Quitting smoking improves HCWs’ trustworthiness in the eyes of patients, and minimizing active smoking in workplaces can deter smokers [31]. Although workplace environments for smoke cessation have a greater success rate and lower dropout rate than clinical settings [32], it is well-known that success usually declines over time. This is probably due to the tangential role relied on the psychological aspect of the dependence [33], which should be overcome combining training courses and psychological support [34].

Previous research documented that the economic burden of smoking on society lies on many factors, including the huge medical care required (up to 15% of total healthcare costs in developed countries), increased morbidity and mortality, and decreased productivity of smokers [33,35]. The primary expenditures associated with worker absenteeism for smokers were attributed to tobacco use in the cost analysis. For instance, a meta-analysis of working adults conducted in the U.K. highlighted a major incidence of SAD between smokers and former smokers (19% increase in risk) and between the latter compared to never smokers (14% increase in risk) [36]. Smoking can interfere with work capacity, contributing to work impairment [37].

From an econometric standpoint, positive evidence of economic returns over investment of worksite-based incentives and competitions to reduce tobacco use among workers has been highlighted in terms of net cost savings to employers (averted healthcare expenses and productivity losses) based on referenced secondary estimates [38]. Our findings expressed a recovered time from smoking, which leads to a recovery in productivity after the SSP intervention. Additionally, since work performance is influenced by a variety of objective factors (e.g., work climate and environment) and subjective factors (e.g., personal relationship of the worker with colleagues and superiors), performance indicators are not reliable for the evaluation of the effectiveness of WHP programs.

Research suggests there are health benefits in reducing the number of cigarettes smoked even without quitting [39]. We collected information about the reduction of cigarettes workers who failed to quit smoked. The small number of cases prompted us not to develop this analysis; however, in the future, when the program is applied extensively, we will also evaluate the economic and health benefits of reducing the number of cigarettes smoked.

In the occupational medicine scenario, this research endorses the effectiveness of WHP initiatives focused on improving the workforce’s health, as previously reported in similar studies conducted on the same working population [40,41]. In the perspective of Total Worker Health ®, the interaction between occupational physicians in the worker-employer relationship is supported by econometric indicators, which serve as tangible evidence of the win-win approach of WHP programs [42,43]. In Italy, this approach is particularly relevant considering the higher smoking rates than workers coming from other countries in healthcare [6,7].

This study has many limitations, which are concatenated with each other. Firstly, the sample was self-selected, resulting in an ineradicable selection bias, since the primary interest of the experimenters was to test the method and verify its applicability as quickly as possible. Consequently, no criteria for admission to the course were defined in advance. The lack of selection of participants meant that the level of workers’ motivation could not be ascertained beforehand. It is possible that some of the participants did not have an actual intention to quit, but still felt that what the course offered was useful for their training. This factor may have influenced the frequency of dropouts. However, in workplaces, the equity criterion dictates that any device or method that may be beneficial to health should be made equally available to all workers.

In a highly mobile population such as hospitals, the loss of observations in the longitudinal study is to be expected as it depends on the exit of many workers from the cohort being monitored within the company. The continuation of the study should include the possibility of continued follow-up of workers, either through their own general practitioners or the occupational physicians of the companies to which they moved to work.

Workers who were not willing to quit smoking did not participate in the course; this deprived the study of a control group. Future replication of initiatives aimed at smoking cessation will allow comparison of the effectiveness of the intervention model tested here with other models, and evaluate the econometric analyses of each one.

The pilot study enrolled a modest number of workers, in view of the small number who could be effectively followed by psychologists in the course. The small sample of the participants represents another pitfall of the study. However, the demonstration of the program’s cost-effectiveness in a small group of workers is an incentive to invest more on WHP initiatives.

The pilot study was conducted only on a small group of workers who had already matured the decision to quit smoking. Due to the promotional intervention’s urgency, it was not preceded by an information campaign on the risks of smoking for all workers, nor by targeted attempts to increase the number of workers involved. However, the rapid implementation of the program has provided results that can be useful in improving knowledge of the risk and demonstrating to workers that participation is easy, free, and effective.

From the strengths of this study, we can consider that the fact that we announced the initiative to all hospital workers meant that all professional groups participated. The publicizing of the course and the publicizing of the positive results helped to make workers aware of the significance of the problem and management’s determination to push for a reduction in smoking habits. Thus, an attempt to generalize the results of the success rate and economic assessment likely corresponds to the hospital’s scenario.

## Conclusions

Our study demonstrates that WHP programs are straightforward, cost-saving tools that may aid in better controlling the smoking epidemic, even in demanding work environments where there is a high risk of psychological impairment, such as healthcare settings [44]. Moreover, the healthcare setting represents a breeding ground for tackling tobacco use among healthcare personnel and spreading beneficial effects of quitting smoking in the community. Future research on larger working communities will deep our findings both in healthcare and non-healthcare settings.

## Data Accessibility Statements

The data presented in this study are available upon request from the corresponding author. The data are not publicly available due to ethical restrictions.
